# *Candida auris*: A Decade of Understanding of an Enigmatic Pathogenic Yeast

**DOI:** 10.3390/jof6010030

**Published:** 2020-02-26

**Authors:** Ryan Kean, Jason Brown, Dolunay Gulmez, Alicia Ware, Gordon Ramage

**Affiliations:** 1 Department of Biological and Biomedical Sciences, School of Health and Life Sciences, Glasgow Caledonian University, Glasgow G4 0BA, UK; 2 School of Medicine, Dentistry and Nursing, College of Medical, Veterinary and Life Sciences, Glasgow G2 3JZ, UK; 3 Medical Microbiology Department, Faculty of Medicine, Hacettepe University, Ankara 06230, Turkey

**Keywords:** *Candida auris*, pathogenicity, in vivo models, biofilms

## Abstract

*Candida auris* is an enigmatic yeast that continues to stimulate interest within the mycology community due its rapid and simultaneous emergence of distinct clades. In the last decade, almost 400 manuscripts have contributed to our understanding of this pathogenic yeast. With dynamic epidemiology, elevated resistance levels and an indication of conserved and unique pathogenic traits, it is unsurprising that it continues to cause clinical concern. This mini-review aims to summarise some of the key attributes of this remarkable pathogenic yeast.

## 1. Introduction

A decade on since its discovery, *Candida auris* has rapidly emerged as a significant nosocomial pathogen, responsible for an escalating number of infections across the globe. *C. auris* possesses some almost unique characteristics for an infectious yeast in that it can survive and persist within the environment for prolonged periods and a significant number of clinical isolates have been reported to be multi-drug resistant, with a very small proportion resistant to three classes of antifungals [1]. The simultaneous emergence of genetically unrelated clades of this organism has sparked thoughts as to how this drug resistant yeast originally manifested, with only just recently theories being proposed on its emergence, linked to some novel biological traits, which will be discussed in this review [2,3].

## 2. Clinical and Epidemiological Basis of Disease

To date, 39 countries have reported documented cases of *C. auris* infection, with clinical case reports from all inhabited continents. The first clinical report was from an isolate from an ear canal in 2009 in Japan [4], with 15 other ear isolates detected in the same year in South Korea [5,6]. These initial reports indicated an uncommon fungal infection that was predominantly limited to ear isolates. In 2011, the first candidemia cases were described in South Korea, one of which dated back to 1996 [7]. Since then, invasive infections, especially candidemia, have increased [8]. The emergence of *C. auris* has changed candidemia epidemiology in certain countries, even outcompeting the most common fungal pathogen *Candida albicans* at a number of centers in South Africa [9,10,11,12,13]. Additionally, outbreaks continuing for months have been described, sometimes resulting in the closing of intensive care units [10,14,15]. Given the initial and continuing difficulty in correctly identifying *C. auris*, the extent and overall burden of the problem might be underestimated [8,16,17,18,19,20].

Perhaps the most fascinating insight into *C. auris* was the discovery of the simultaneous emergence of genetically unrelated clonal populations across three different continents. This pioneering discovery from Lockhart and colleagues revealed through whole genome sequencing that four independent clades existed. These clades appeared to be geographically specific and are commonly classified as South Asian (clade I), East Asian (clade II), African (clade III), and South American (clade IV) clades, and differ by tens of thousands of single nucleotide polymorphisms (SNPs), with minimal genetic intra-clade differences (<60 SNPs) [1]. Interestingly, a recent study has suggested a potential fifth *C. auris* clade from an ear swab isolate of an Iranian patient. The patient had no documented travel history, suggesting that this emergence of *C. auris* may have not been a recent introduction. Despite being pathologically similar to the East Asian clade, this isolate was hundreds of thousand SNPs different than this and the other three clades [21]. The stimuli for the unique emergence of *C. auris* are not well understood, with suggestions of anthropogenic factors playing a contributing role.

## 3. Is *Candida auris* a Virulent Pathogen?

As our clinical knowledge of *C. auris* has expanded, we have sought in parallel to understand why this yeast is portrayed as such a successful pathogen. With a somewhat limited molecular understanding of *C. auris*, details are now beginning to emerge in the literature to shine a light on key elements of its virulence profile.

The first description resembling the basis of a virulent phenotype appeared in 2015, when simple experimental approaches described the expression of phospholipase, proteinase and hemolysin activity [22]. Next, some more detailed studies from Borman and colleagues (2016) reported differential growth rates and release of daughter cells that manifested itself as an aggregating phenotype, which was less virulent than non-aggregating cells [23]. This study was the foundation for the first definitive study detailing its ability to differentially form antifungal tolerant biofilms [24]. Interestingly, a more recent study has identified that cellular aggregation is in fact an inducible phenomenon that can be triggered by sub-inhibitory concentrations of triazole and echinocandin antifungals [25]. Collectively, these laboratory studies, and confirmatory analyses [26], highlighted the pathogenic potential of *C. auris*. Moreover, utilising *C. albicans* as a comparator species has helped improve our overall understanding and expand our capabilities to investigate this resilient and tolerant yeast [27,28].

An advantage of *C. auris* coming ‘late to the party’ is that genomic technologies and the use of comparator species have supported finding orthologues of proteins well-characterised in *C. albicans*. The first study to do so came from India, where Chaterjee and colleagues (2015) reported that it shared significant virulence attributes, including mannosyl transfersases, oligopeptide transporters, secreted proteases and genes involved in biofilm formation [29]. These observations support the aforementioned laboratory studies; however, a limitation to these data are that many genes encoded hypothetical and undefined proteins. How do we determine the functionality and relative importance of these, or do we simply discount these? More robust studies that have followed substantiated these observations, including multiple genes encoding drug transporters, secreted aspartyl proteinases and lipases [30]. One way this has been approached is through using transcriptomics data sets. We were able to undertake a de novo transcriptome assembly that was assembled into an approximately 11.5Mb transcriptome, consisting of 5848 genes [31]. Here, a number of important transcripts were differentially expressed, particularly adhesins IFF4 and CSA1, ALS-gene family members, and other biofilm related genes, such as secreted aspartyl protease and extra-cellular matrix related components. More recently, it has been reported through the use of multi-omics approaches that in comparison to *C. albicans*, *C. auris* exhibits significant differences in carbon utilisation and downstream cellular protein and lipid content [32]. It is likely that further pathogenic insights will begin to emerge as large scale data-sets are produced. This intensified effort will enable us to fully comprehend why this yeast can be so damaging to patients.

## 4. Stress Responses and Key Signaling Pathways of *Candida auris*

Survival strategies and adaptation to multiple ecological niches are prerequisites for many successful pathogens. Some of the defining features of *C. auris* are its high temperature and salt tolerance, which can permit survival in harsh environments [33]. At 37 °C and 40 °C, *C. auris* grows comparatively with *C. albicans*; temperatures at which the more genetically similar *Candida haemulonii* struggles, or cannot grow. Additionally, a number of *C. auris* isolates have been shown to grow at temperatures as high as 42 °C, which inhibits *C. albicans* growth [34]. This organism also demonstrates high salinity tolerance, growing at 10% *wt*/*vol*, a potential factor in being able to quickly and more readily detect and culture this pathogen [35]. Interestingly, these elevated salt concentrations can induce the formation of elongated cell morphologies, similar to pseudohyphae of other *Candida spp.* [36]. However, the biological mechanisms and significance of this morphogenic switching remain to be elucidated.

Adaptation to environmental and host responses are typically controlled by a variety of different, conserved signaling pathways across many pathogenic fungi. Studies understanding the functions of such pathways in *C. auris* are still well within their infancy; however, some studies have begun to elucidate these functions. One such key stress response is the Hog1-related stress-activated protein kinase (SAPK) signaling pathway. Using *hog1Δ* strains, Day et al. (2018) demonstrated multiple functions for SAPK in resistance to environmental stressors such as H_2_O_2_ and osmotic stress [37]. Interestingly, the *hog1Δ* strain displayed attenuated virulence compared to the wild type, which could be as a result of the mutant forming large aggregates, which has been observed in clinical isolates that are less virulent than non-aggregating counterparts [23,24]. In line with these previous studies on aggregation, the SAPK pathway could be significant in the formation of aggregates as a potential survival strategy. Other key stress response pathways are dependent on the protein phosphatase calcineurin, which interacts with the molecular chaperone protein HSP90. Analysis of protein function of Hsp90 in *C. auris* has been shown to have multiple roles and essential for growth [38]. Interestingly, Hsp90 was shown to negatively regulate filamentation in *C. auris*, with inhibition of Hsp90 resulting in elongated pseudohyphae-like structures, morphologically similar to those induced by high salinity and passage through mammalian hosts [36,39]. Furthermore, its inhibition was shown to increase fluconazole susceptibility of two *C. auris* isolates to which did not harbor any common *ERG11* mutations related to azole resistance [38].

The organisms’ ability to survive in these typically microbe-unfavorable conditions are said to be key factors in the recently proposed climate change hypothesis by Casadevall and colleagues, and its transition from being a previously unknown environmental yeast to a significant nosocomial pathogen, with its potential intermediate transmission through an avian host [2]. The authors of this study provide a tantalising hypothesis that higher ambient environmental changes brought about by human intervention has led to the selection of thermally tolerant fungal species that can infringe upon the protective thermal restriction zone. The thermal restriction zone, i.e., the temperature differential between the high protective basal temperatures of mammals and that of ambient environmental temperatures, may be narrowing as a result of climate change. Casadevall and colleagues (2019) undertook comparative analysis of the *C. auris* and closely related relative species, demonstrating that these were not generally thermotolerant to mammalian temperatures [2], indicating that they have not evolved as human pathogens. We know that *C. auris* constitutively overexpresses heat shock protein 90, and by borrowing knowledge from other *Candida* pathogens we can surmise that this may account for its virulence, thermal tolerance, osmotic-stress tolerance and multidrug resistance. These ideas taken together, and with caution until we have definitive evidence, allow us to postulate that *C. auris* is an environmental yeast that has made the opportunistic leap to human hosts. It preferentially locates and persists on the cooler and salty environments of the skin, even with the capacity to form tolerant biofilms structures [40]. Intriguingly, could it be that aqueous and salty environments of wetlands and marshes are more prone to climatic change, and this has propelled *C. auris* success? Alternatively, could the drivers for this emergent pathogen have a simpler explanation, propelled by an ecotoxicological threat of high-level of azole antifungals found in our own wastewater [41]? Irrespectively, whether one or a combination of these theories is correct, it is highly likely that the emergence of *C. auris* is a human-made phenomenon.

## 5. Biofilms and Surfaces

A key pathogenicity mechanism of various *Candida spp.* is the formation of highly organised and structured communities known as biofilms. The ability of clinical isolates to form biofilms has been identified as a significant risk factor negatively associated with patient outcomes for both *C. albicans* and *Candida parapsilosis* [42,43], as well as other species, such as *Candida glabrata*, frequently isolated from indwelling medical devices [44]. To date, there are limited studies detailing the clinical significance of *C. auris* biofilms with regards to risk factors for invasive disease. Isolates have however been recovered from a number of clinical sites including wounds, stents, temperature probes and central lines, suggestive of a biofilm-related lifestyle in host [14,34,45].

Biofilm formation was initially disregarded in a study using isolates from the East Asian clade [6]. Interestingly, this clade appears to display some unique behavioral patterns in comparison to other clades, including lower fluconazole resistance rates and high incidence of causing otitis [46]. In addition, these isolates rarely cause invasive infections and large-scale outbreaks, which could explain the inability of these organisms to form biofilms [47]. A number of studies have started to look at the implications of *C. auris* biofilms, particularly with regards to drug tolerance [31,48,49,50]. These studies have shown that various *C. auris* isolates can selectively tolerate clinically significant concentrations of all three classes of antifungals.

Biofilm tolerance mechanisms employed by this emerging pathogen appear analogous as to those of *C. albicans*, the primary biofilm forming pathogen within the genus. Using a temporal transcriptomic analysis of various stages of biofilm, our group characterised the key processes which govern biofilm formation and its associated increased antifungal tolerance. Efflux pumps of both ATP binding cassette and major facilitator superfamily transporters, were up-regulated as the biofilm matured to 24 hours of growth compared to planktonic cells [31]. It remains unknown as to whether a *C. auris* biofilm cell is more virulent compared to an equivalent planktonic cell, as was shown with *C. albicans* [51]. Given that gene expression of the hydrolytic enzymes PLB3 and SAP5 were also up-regulated in biofilm cells within our data-set, then it would be plausible that they indeed may be more virulent and invasive [31]. Another key component of fungal biofilms is the formation of the extracellular matrix (ECM) [52]. Like other biofilm tolerance mechanisms, the composition and function of the *C. auris* biofilm matrix appears to be conserved with other *Candida spp.* [53]. Using radiolabelled fluconazole, the authors demonstrated that this protective substrate is able to sequester between 50% and 90% of available fluconazole within the matrix, rendering the drug unable to reach its target. In addition, physical removal and chemical degradation of individual polysaccharides of the matrix significantly increasing the susceptibility of these communities to fluconazole [53]. Under biofilm-inducing conditions, mass-spectrometry analysis identified significant quantities of tyrosol in the supernatant, a quorum sensing molecule involved in *C. albicans* biofilm formation, suggesting another potentially conserved biofilm function [54].

In addition to reducing the susceptibility to antifungal drugs, it has been speculated that *C. auris* biofilms may have an environmental function in facilitating nosocomial survival and persistence. The formation of environmental biofilms has been proposed as survival mechanism for a number of microbial pathogens, most notably *Staphylococcus aureus* [55]. These communities are thought to play a key role in hospital acquired infections and can remain viable for multiple weeks, tolerate commonly used disinfection procedures and can subsequently transmit to other surfaces. In laboratory-based studies, *C. auris* has demonstrated all of these characteristics surviving on various abiotic substrates such as plastic and steel as long as four weeks [35] and also being recovered for multiple different fomites in the hospital environments [56]. *C. auris* demonstrates variable susceptibility to a number of disinfection reagents (reviewed in [57]) with studies only recently beginning to understand the effects of disinfectants against surface-adherent *C. auris* cells. Using a *C. auris* dry-surface biofilm model, Ledwoch and Maillard demonstrated that these communities can remain viable after certain biocide treatment, and are also able to successfully regrow and transfer to another sterile substrate post disinfection [58]. In addition, Short et al. (2019) showed the up-regulation of biofilm-related genes after 14 days of environmental survival, including those involved in adherence, drug resistance and ECM formation [59].

The emerging data in the field appear to support the notion that biofilm biology is an important aspect of *C. auris* lifestyle and going forward this direction of research will likely provide important details on its persistence and antifungal tolerance. Indeed, it may also expose some clues to support the notion that *C. auris* is a man-made phenomenon.

## 6. In vivo Models of Invasive Candidiasis to Study Host–*C. auris* Interactions

**Figure 1:**
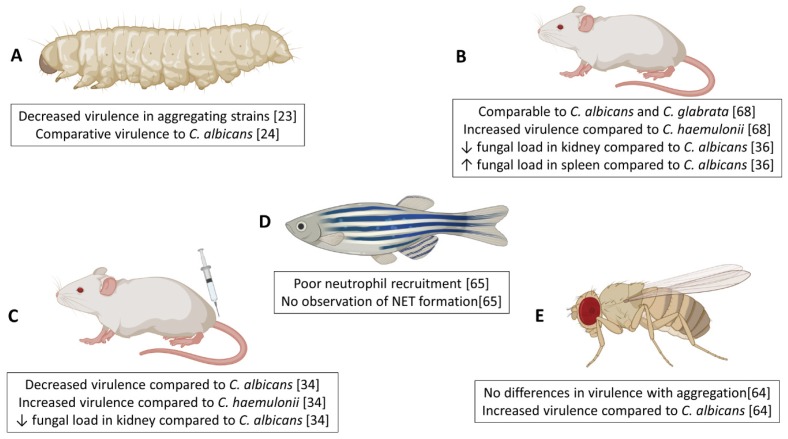
Summary of in vivo pathogenicity studies of *Candida auris.* The virulence of *C. auris* in comparison to various other *Candida spp.* has been assessed in a number of different vertebrate and invertebrate systems including *Galleria mellonella* (**A**), immunocompetent mice (**B**), immunocompromised mice (**C**), *Danio rerio* (**D**) and *Drosophila melanogaster* (**E**). Figure was created with BioRender.

Integral to understanding the virulence of microbial pathogens is the successful utlisisation of in vivo models. Several groups have investigated the pathogenicity of *Candida auris* in a variety of in vivo systems, including invertebrate, fish, and murine models, with key and sometimes conflicting findings highlighted in Figure 1. Initial studies were performed by Borman and colleagues, using the invertebrate moth larvae (*Galleria mellonella*) model of systemic candidiasis, which was used to explore the virulence traits of non-aggregating and aggregating forms of *C. auris* in vivo [23]. In this system, *C. auris* was found to be as virulent as *Candida albicans* and *Candida tropicalis*. This high virulence rate is especially important given that *C. auris* strains do not produce hyphae comparable to that of other *Candida spp.* [23]. Our group have corroborated such findings within this *G. mellonella* model, identifying that two non-aggregative *C. auris* strains possessed enhanced virulence capacity over aggregating counterparts, as well as *C. albicans* at reduced inoculum concentrations [24]. *G. mellonella* provide useful model platforms due to reduced ethical implications, whilst also possessing different components of the innate immune system comparable to the mammalian system (reviewed in Trevijano-Contador et al., 2018 [60]). Studies have used these models to explore the early cellular and humoral responses in *G. mellonella* following infection with *C. albicans* [61,62]. Of note, a study by Sheehan and Kavanagh (2018) identified a bisphasic response by the larvae to *C. albicans* infection; early response (>6 hours) is driven by non-specific immune reactions (antimicrobial peptides release and melanisation) whilst later stages are characterized by specific responses (increases in haemocyte number resulting in larval death) [62]. To our knowledge, studies have yet to examine the host response in *G. mellonella* to *C. auris*. Future work may warrant such investigations, to fully elucidate mechanisms by which *C. auris* phenotype is driving differential levels of virulence in this model, with emphasis on histological changes, alterations in haemocyte number and gene or protein expression of key host molecules, such as antimicrobial peptides, opsonins and lysozymes.

Another invertebrate organism using to study infectious disease is *Drosophila melanogaster*, which has been previously used with fungal pathogens including *Candida albicans* [63]. Using Toll-deficient (*Tl*) *D. melanogaster* flies, Wurster et al. (2019) demonstrated that 10 *C. auris* isolates representing all four geographical clades were significantly more virulent than a *C. albicans* isolate [64]. Interestingly, and in disagreement with previous studies [23,24], the aggregative capacity of isolates had no effect on pathogenicity in *D. melanogaster* to which the authors hypothesise to be due to the impaired phagocytic and immune function in *Tl*-deficient flies.

A model of invasive candidiasis using a zebrafish (*Danio rerio*) system has recently been used to visualize the host-pathogen interactions between neutrophils and *C. auris* in vivo [65]. These in vivo systems hold many advantages over invertebrate models, including close similarity in genetics, physiology, anatomical structure, and innate and adaptive immune functions with mammalian hosts [66]. A number of publications have used the zebrafish model to investigate the roles of macrophages and neutrophils in immune defense against a range of fungal species including *Aspergillus*, *Candida* and *Cryptococcus* species (reviewed in [67]). Moving forward, examination of the zebrafish host response to multiple *C. auris* strains from various clades with diverse phenotypes is necessary to fully elucidate any potential interactions between different *C. auris* strains and host cells in vivo.

Recent studies have documented the virulence of *C. auris* in murine models of invasive candidiasis. In a similar manner to observations from *G. mellonella* models, survival rates in mice post-infection with *C. auris* are strain-dependent [34,36,68,69], further affirming that genetic variability among the strains collected from diverse geographic locations impacts the organisms virulence. Two comparative virulence studies for multiple *Candida* species used an immunocompromised murine model to show that two *C. auris* isolates from India, and one isolate from Israel, displayed similar virulence to *C. albicans* strains [34,68]. Conversely, a *C. auris* isolate from China was non-virulent in a BALB/c mouse with normal immune system, whilst all *C. albicans*-infected mice died within six days of infection [36]. Many fungal pathogens including *C. auris* disproportionally affect immunocompromised patients within healthcare settings and as such it is important to characterise host-pathogen interactions in models that mimic real-life circumstances. In a study by Xin et al. (2019) it was shown that immunocompromised mice were slightly more susceptible to *C. auris* infection than immunocompetent mice [69], suggesting a crucial role for a healthy immune system in controlling *C. auris* virulence. A fascinating observation from histopathological analyses in these candidiasis murine studies was that *C. auris* accumulated in the kidney of the mice in the form of aggregates [34,39,68], potentially offering an explanation for the survival and persistence of the organism in vivo. In addition, a study by Yue et al. reported that a small percentage of yeast cells isolated from murine liver and kidney tissue displayed a filamentous phenotype morphologically similar to hyphae in other *Candida spp.* when cultured ex vivo at a low temperature [39]. Passage through a second murine host revealed that filamentous cells switched to ‘filamentous competent’ yeast in vivo, but maintained their filamentous phenotype ex vivo. Tissue burden studies revealed a higher fungal load within the brain and lungs of mice infected with filamentous cells than typical yeast cells, with a skin colonisation assay revealing that filamentous cells were capable of invading the underlying epidermal tissue of mice with topical skin infection, compared to superficial colonisation by typical yeast cells [39]. More recently, a porcine skin model has been reported, where it was shown that *C. auris* had the capacity to proliferate and form multilayer biofilm communities [40]. This is some of the most compelling evidence of how it may survive and persist in healthcare environments. Further studies are required to assess the host response in tissues and systemically following *C. auris* infection.

Given the well-documented antifungal resistance rates and limited therapeutic options, murine infection models provide key data to better inform prescribing practices for clinical *C. auris* cases. Using neutropenic mice, Lepak et al. (2017) performed pharmacodynamics studies on nine *C. auris* isolates from representative geographical clades and demonstrated the cidal effect of micafungin against tested strains, highlighting its use as the first-line therapy for infected patients [70].

The use of arthropod, mammalian and invertebrate models in studying *C. auris* infection from each of the various geographical clades has greatly enhanced our understanding of the virulence and pathology of this emerging pathogen. There still remains a number of answered questions including the underlying biological processes responsible for observed differences in virulence between isolates from the varying geographic clades.

## 7. Hide and Seek—Immune Evasion or Lack of Immune Surveillance?

**Figure 2:**
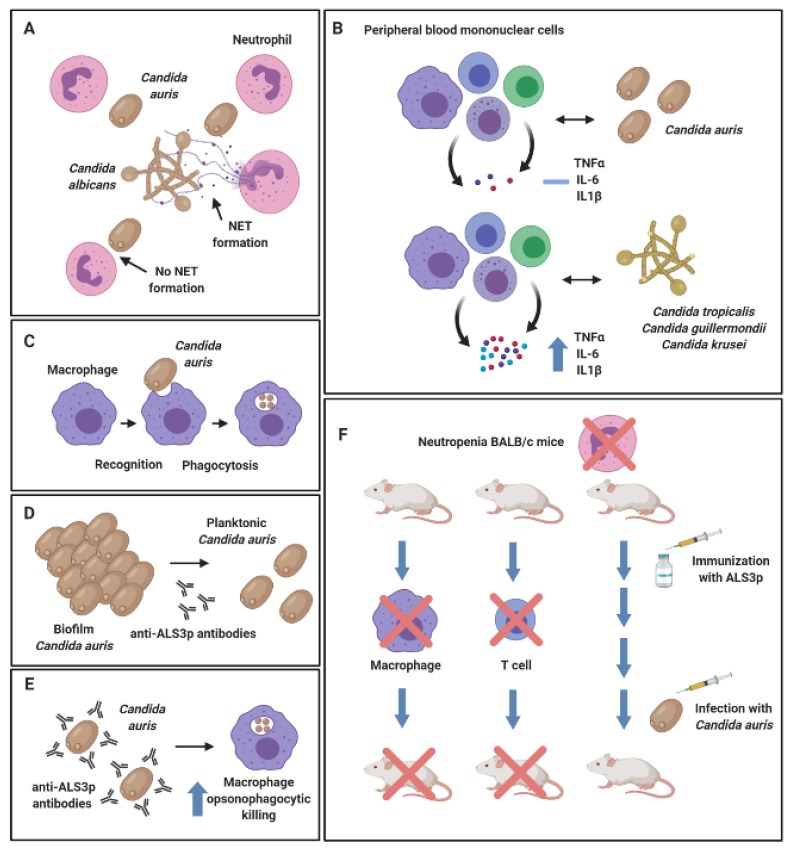
Host-pathogen interactions of *Candida auris. Candida auris* evades neutrophil capture via neutrophil extracellular trap (NET) formation. Neutrophils preferentially target *Candida albicans* in mixed cultures with *C. auris* (**A**). Peripheral blood mononuclear cells (PBMCs) fail to induce a potent pro-inflammatory cytokine response against *C. auris*, whilst *Candida tropicalis*, *Candida guilliermondii* and *Candida krusei* induced production of TNF-α, IL-6 and IL1-β in PBMC co-cultures (**B**). Human monocyte-derived macrophages were shown to recognize and phagocytose *C. auris* (**C**). Anti-Als3p antibodies generated by the NDV-3A vaccine in mice significantly reduced biofilm formation capabilities in *C. auris* (**D**) and enhanced opsonophagocytic killing by murine macrophages in vitro (**E**). Neutropenic mice susceptible to lethal *C. auris*-disseminated infection were protected following Als3p vaccination. Depletion of macrophages and CD4+ T cells in this model resulted in reduced survival rates in mice, suggestive that these cell subsets play an important role in NDV-3A vaccine-mediated protection (**F**). Figure was created with BioRender.

From the in vivo studies described above, it could be postulated that *C. auris* uses sophisticated mechanisms to evade the immune response and thereby escape expulsion from the host. Indeed, the work of Johnson et al. (2018) showed that *C. auris* were resistant to neutrophil-mediated killing in vitro, whilst neutrophils preferentially targeted and engulfed *C. albicans* in mixed cultures with *C. auris* (Figure 2A). In the zebrafish model, 50% less neutrophils were recruited, and a reduced number of neutrophil extracellular traps were produced in response to *C. auris* over *C. albicans* infection [65]. Another study showed that viable *C. auris* failed to induce a significant inflammatory response in human peripheral blood mononuclear cells (PBMCs), whilst live *Candida tropicalis*, *Candida guilliermondii* and *Candida krusei* induced a much greater release of TNF-α, IL-6 and IL1-β (Figure 2B). Conversely, heat-killed organisms, including *C. auris*, stimulated an elevated production of all pro-inflammatory mediators [71]. The authors postulated that heat-inactivation resulted in artificial exposure of chitin and β1,3-glucan on the surface of the cell wall, leading to a dectin-1 receptor stimulation and an elevated immune response as previously described with *C. albicans* [72]. This hypothesis may suggest that the morphological structure and/or aggregative phenotype of *C. auris* could dictate its immunomodulatory effects. Moreover, the same study showed that human monocyte-derived macrophages were shown to interact with and phagocytose *C. auris* (Figure 2C), yet not to the same efficiency as other *Candida* species, such as *C. tropicalis*, *C. guilliermondii* and *C. krusei*. Therefore, it can be concluded from this study that the host recognizes the organism (e.g., macrophage recognition), yet fails to establish an effective immune response against a viable *C. auris* cell (an elevated pro-inflammatory cytokine response).

Interestingly, a publication by Singh et al. (2019) elegantly showed that vaccination of neutropenic BALB/c mice with NDV-3A (a vaccine targeting the N-terminus of Als-3 protein formulated with alum) recognized *C. auris* in vitro and protected immunosuppressed mice from otherwise lethal *C. auris*-disseminated infection [73]. In vitro, the authors found that anti-Als3p antibodies generated by the NDV-3A vaccine in the murine model inhibited biofilm formation (Figure 2D) and enhanced the opsonophagocytic killing of *C. auris* by macrophages (Figure 2E). In vivo, an important functional role for macrophage and CD4+ T cells was identified in the NDV-3A vaccination mechanism, whereby depletion of these immune cells in the murine model compromised the NDV-3A vaccine-mediated protection against *C. auris* (Figure 2F) [73]. This is further evidence that *C. auris* is recognised by the immune system under certain physiological conditions. At the time of writing, investigations into host-pathogen interactions in *C. auris*-mediated infection are limited; future studies must continue to explore the effects of various *C. auris* strains on different subsets of the innate and adaptive immune system, both in vitro and in vivo.

## 8. Concluding Remarks

*C. auris* demonstrates pathogenic traits of a successful pathogen. Undoubtedly, antifungal resistance is the most significant benefit to this yeast, and when combined with thermotolerant and biofilm attributes, then its future importance to clinical mycology remains undisputed. Whether further distinct clades emerge from different geographical regions remains to be seen, though we are better prepared a decade later in our ability to react and manage these infections. The next ten years will provide opportunities to develop a greater understanding of *C. auris* cell biology and how it interacts with host immunity. Moreover, as new classes of antifungals come through the pipeline, then novel therapies will offer some optimism for control of this yeast. Additionally, as next generation sequencing technologies improve, we will enhance our ability to carefully map interspecies and interkingdom interactions. This will enable us to determine whether *C. auris* synergises or is antagonized by co-colonising microorganisms. This may form a platform for alternative bio-control opportunities as suggested by recent probiotic studies [74].

Going forward, we must be cautious as a community not to lose perspective on the key issues in medical mycology. To a large extent, *C. auris* represents an unknown, and this has led to an intensified focus to understand this emergent pathogen. There are significant amounts of low hanging fruit for the taking, but this should not be to the detriment of understanding more important mycological issues, such as antifungal resistance. Compared to other *Candida* species, *C. auris* does not appear to have the same extensive global footprint and burden on human health, and it does not have the same arsenal of virulence attributes. As it stands, it remains worthy of our consideration, but whether it continues to dominate our attention, or simply becomes a footnote in our history, remains to be seen. It is difficult to predict the future for this enigmatic yeast, but we can be sure that it will preoccupy the mycology research community for the next decade.
